# Neural Correlates of Migration: Activation of Hypothalamic Clock(s) in and out of Migratory State in the Blackheaded Bunting *(Emberiza melanocephala)*

**DOI:** 10.1371/journal.pone.0070065

**Published:** 2013-10-21

**Authors:** Ashutosh Rastogi, Yatinesh Kumari, Sangeeta Rani, Vinod Kumar

**Affiliations:** 1 Department of Zoology, University of Lucknow, Lucknow, India; 2 Department of Zoology, University of Delhi, Delhi, India; University of Rouen, France

## Abstract

**Background:**

Many vertebrates distinguish between short and long day lengths using suprachiasmatic nuclei (SCN). In birds particular, the mediobasal hypothalamus (MBH) is suggested to be involved in the timing of seasonal reproduction. This study investigated the response of SCN and MBH to a single long day, and the role of MBH in induction of the migratory phenotype in night-migratory blackheaded buntings.

**Methodology/Principal Findings:**

Experiment 1 immunocytochemically measured c-fos in the SCN, and c-fos, vasoactive intestinal peptide (VIP) and neuropeptide Y (NPY) in the MBH of buntings exposed to a 20 h light period. Long light period induced significantly stronger c-fos expression, measured as number of c-fos-like immunoreactive (c-fos-lir) cells, in MBH, but not in the SCN. Within the MBH, c-fos-lir cells were significantly denser in the inferior hypothalamic nucleus (IH) and infundibular nucleus (IN), but not in the dorsomedial hypothalamus (DMH). IH and IN also had significantly increased number of VIP and NPY labeled cells. DMH had significantly increased number of VIP labeled cells only. Experiment 2 assayed c-fos, VIP and NPY immunoreactivities in the middle of day and night in the MBH of buntings, after seven long days (day active, non-migratory state) and after seven days of *Zugunruhe* (night active, migratory state) in long days. In the migratory state, the number of c-fos-lir cells was significantly greater only in DMH; VIP-lir cells were denser in all three MBH regions suggesting enhanced light sensitivity at night. The denser NPY-lir cells only in IN in the non-migratory state were probably due to premigratory hyperphagia.

**Conclusions/Significance:**

In buntings, SCN may not be involved in the photoperiod-induced seasonal responses. MBH contains the seasonal clock sensitive to day length. VIP and NPY are parts of the neuroendocrine mechanism(s) involved, respectively, in sensing and translating the photoperiodic message in a seasonal response.

## Introduction

It is suggested that the self-sustained endogenously generated circannual rhythms (*circa* = about; *annum* = year) control annual (seasonal) life history processes like breeding and migration cycles in birds [1], [2]. In close interaction with external cues (e.g. day length), circannual rhythms provide seasonal timing, in synchrony with the yearly calendar time [2], [3]. Interestingly, the mechanism by which a seasonal species distinguishes between changing day lengths in the year involves circadian (circadian, *circa* = about; *dian* = day) rhythms [2]–[4]. Each day, a photoperiodic species shows circadian rhythm of sensitivity to light, which in interaction with daylight induces a specific response. Thus, circannual rhythms are involved in the seasonal timing [1], [5], while circadian rhythms are involved in the maintenance, on a daily basis, of the seasonally timed behavior or physiology [6]–[8].

In mammals, the principal circadian pacemaker lies in the suprachiasmatic nucleus (SCN), which responds to day length and functions both, as the daily and seasonal clock [9]. In birds, on the other hand, circadian pacemaker system is present at a minimum of three separate clock sites, the retina of the eyes, the pineal gland and the hypothalamus [2]. Unlike in mammals, the avian SCN consists of two neuronal populations, mSCN (medial SCN, does not receive light input from the retina) and vSCN (visual SCN, receives light input from the retina) [10]. These three circadian clocks interact and regulate daily activity-rest cycle [2]. Further, it is known that these three clocks may not directly regulate photoperiodic response of the hypothalamo-piutitary-gonad (HPG) axis [2], [11], [12], but it is unclear whether they have a role in regulating other seasonal events. There is no direct evidence that these clocks are not involved in avian migration. However, there is evidence of the involvement of circadian clocks in control of the phenologies linked with seasonal reproduction (gonadal maturation) and migration (viz. body fattening and premigratory night time restlessness, *Zugunruhe*) [6]–[8], [13].

Several lines of evidence including those from the lesion and electrical stimulation studies, suggest mediobasal hypothalamus (MBH) in birds as the photoperiodic induction site [14], [15]. Using c-fos (the protein product of *c-fos*, a proto-oncogene) expression as an indicator of the neural activity, studies have shown that MBH rapidly responds to light [12], [16]. At the end of the first long day, the response is measurable both at the levels of genes (clock genes: *period 2*, *cryptochromes*, *clock* and *bmal1* [17], *Eya3* and *Tshβ* [18], [19]; genes encoding thyroid hormone activating and deactivating enzymes, *type 2* and *type 3 iodothyronine deiodinase*, *Dio2* and *Dio3* [18], [20], [21], [22]) and peptide (vasoactive intestinal peptide, VIP [21]) and proteins (e.g. gonadotrophin releasing hormone, GnRH [23]).

The MBH contains vasoactive intestinal peptide (VIP) secreting neurons, which have been suggested as the encephalic photoreceptors (EPRs) [21], [24]. The MBH possibly communicates with the lateral septal organ (LSO), which also contains light sensitive neurons, the EPRs [25]. There is a direct neuronal projection from VIP containing CSF-contacting neurons in the LSO to median eminence (ME) [21]. In the LSO, VIP immunoreactive (ir) cells were found co-existed with GnRH-I-ir cells in Japanese quail (*Coturnix coturnix japonica*) [26]. VIP is also found involved in prolactin release from the pituitary, both *in vivo* and *in vitro* [27], [28]. Thus, VIP seems to be involved in induction of the photoperiodic responses linked with reproduction [26] including the incubation behaviour [29].

There is also a close association of neuropeptide Y (NPY) with GnRH perikarya in the bed nucleus of pallial commissure (nCPa), infundibular nucleus (IN) and median eminence in birds [30]. This suggests a functional interaction between the NPY and GnRH in regulation of gonadal functions in birds [30]. NPY is reported to be involved in GnRH release [31], gonadal development and sexual maturation [32]. Besides, NPY plays major role in regulating premigratory hyperphagia and fat deposition in migratory birds [33]–[34], and in parental hyperphagia and gonadal activation in non-migratory ringdoves, *Streptopelia risoria* [35]. However, the neural basis of migration is less understood, except a few studies have identified brain areas involved in processes linked with migration, e.g. vision, magnetoreception and olfaction [8], [36]–[38].

To gain insight into how putative daily and seasonal clock sites (the SCN and MBH) in a songbird species acutely responding to stimulatory long days, first we examined the c-fos expression in the SCN and MBH of birds on the first day of transition from short to long days. Then, VIP and NPY were immunohistochemically labeled in the MBH, and their expression was considered as functional correlate of the response of MBH to acute photostimulation. We further compared c-fos, VIP and NPY expression levels in the MBH between non-migratory and migratory states in birds subjected to long days (16 h light∶ 8 h darkness, LD 16/8) and sampled in the middle of day and night first after seven long days, when they were still day active (non-migratory state), and then after birds have shown seven days of night time restlessness (*Zugunruhe*, migratory state). The experiments were done on the highly photosensitive, night-migratory songbird species, the blackheaded bunting (*Emberiza melanocephala*). Buntings show rapid rise in plasma luteinizing hormone (LH) levels on the first long day, i.e. 18 h after the light exposure [39]. Under long days, buntings exhibit migratory phenotypes with phase inversion of neural activity in the olfactory and visual systems in parallel with the behavioral shift, from day to night activity [7], [8]. The results show that MBH contains the photoperiod sensitive seasonal clock, and VIP and NPY are the part of the neuroendocrine mechanism(s) involved, respectively, in sensing and translating the photoperiodic message.

## Materials and Methods

### Animal and experiments

The experiments were carried out as per approval by the Institutional Animal Ethics Committee (IAEC), Department of Zoology, University of Lucknow, Lucknow, India; this is equivalent of the Institutional Animal Care and Use Committee (IACUC) in other countries. The experiments were done on the blackheaded bunting (*Emberiza melanocephala*), which is a Palaearctic-Indian latitudinal long-distance night-migratory songbird species. It arrives in India (∼25°N) in the fall, overwinters, and returns to its breeding grounds situated in the west Asia and south east Europe (∼40°N) in spring [40]. Buntings are not an endangered or protected species. In February, birds were captured from an area located outside of any protected or privately owned area, and as per permission accorded for the purpose of scientific research by Chief Wildlife Warden of the State of Uttar Pradesh, India, where the study was done.

After capture, birds were kept in the outdoor aviary under natural light and temperature conditions (NDL). Then, buntings were brought indoors, and maintained on short days (8 h light∶ 16 h darkness, LD 8/16) at constant temperature (24.0±2.0°C) until the beginning of the experiment. Buntings on short days are unstimulated, but on exposure to long days (>12 h light per day) they exhibit phenotypes linked with migration (body fattening and intense night activity, *Zugunruhe*) and reproduction (gonadal maturation) [7]. Thus, those maintained on short days remain sensitive to light stimulation, and are called photosensitive. The general experimental setup was similar to that described in our recent publication [8]. Briefly, buntings were singly housed in custom designed activity cages placed inside photoperiodic chambers lit by the compact fluorescent tubes (14 watt, Phillips) on a programmed light-dark cycle (LD; L = 150±5.0 lux; D = <1.0 lux). The movement of an individual within its cage was continuously counted through an infrared sensor mounted on the cage, and the activity in 2 min bins was stored using the Stanford Software System, Stanford, CA, USA [41].

### Experiment 1: Effect of transition from short to long day

This experiment examined neural activation of the putative daily and seasonal clock sites, the SCN and MBH, respectively, in response to a single long day exposure, using an experimental paradigm, based on “first day release” protocol employed by Meddle and Follett [16]. We also examined the expression of VIP and NPY as functional correlate of the response of MBH to acute photostimulation. Briefly, a group of twelve unstimulated males maintained on short days (body mass = 25–27 g; testis volume = 0.33–0.45 mm^3^) were singly housed in the activity cages and exposed to short days (8 h light∶ 16 h darkness, LD 8/16). On day 5, six birds were perfused 4 h after lights on (ZT4; zeitgeber time, ZT0 = lights on). For the remaining six individuals, light was not turned off at ZT8 and extended until ZT20 when they were perfused. There was no marked change in the body mass and testis size in 5 days of short days in activity cages.

### Experiment 2: Effect of transition from non-migratory to migratory state

In order to evaluate the role of MBH in induction of the migratory phenotype, this experiment examined protein expression of c-fos, VIP and NPY in the MBH in the middle of the day and night during both the non-migratory and migratory states. A total of 23 unstimulated buntings (group A; n = 11; group B; n = 12; body mass = 25–27 g; testis volume = 0.33–0.52 mm^3^) were used. Individually housed birds in activity cages and placed on short days (LD 8/16) were exposed to long days (LD 16/8). After one week of long days, a group of birds were perfused in the middle of day (ZT8; n = 7) or night (ZT20; n = 4). At this time birds were still day active, hence represented the ‘non-migratory state’. After about two weeks of exposure birds began showing *Zugunruhe*; this was called the onset of the ‘migratory state’. After seven nights of *Zugunruhe*, birds were perfused in the middle of the day or night (n = 6 each time).

### Tissue preparation

We used the protocol, as described in Rastogi et al. [8]. Briefly, deeply anesthetized birds with ketamine - xylazine solution (0.003 ml/g body weight) were transcardially perfused successively with 50 ml saline (pH 7.4) and 30 ml fixative (4% paraformaldehyde in 0.1 M phosphate buffer, pH 7.4). The nighttime perfusions were done under non-stimulatory dim green light at the intensity of <1.0 lux, equal to that of the dark time illumination of an LD cycle. Quickly dissected brains were post-fixed overnight in the same fixative, and thereafter cryoprotected in 10-, 20- and 30% sucrose (Merck) solutions at 4°C. Finally, brains were embedded in 15% polyvinylpyrrolidone (PVP; PVP40T, Sigma) and sectioned in the coronal plane at 30 µm thickness on a Leica CM 1850 cryostat. The four successive sections were separately collected in the phosphate buffer saline (PBS; 10 mM, pH 7.4) and the fifth section was thaw-mounted onto a poly-L-lysine-coated slide. Thus, we had a total of 5 bins with sections separated by 150 µm. We stored first four bins at 4°C until processing for the immunohistochemistry of c-fos, NPY or VIP. The fifth bin sections on slides were processed for Nissl staining [42] to identify anatomical demarcations of suprachiasmatic nucleus, SCN (medial SCN, mSCN; visual SCN, vSCN) and mediobasal hypothalamus, MBH (dorsal tuberal division-dorsomedial hypothalamus, DMH; ventral tuberal division-inferior hypothalamic nucleus, IH and infundibular nucleus, IN) as per descriptions in Cantwell and Cassone [10], Yoshimura et al. [20] and Sharp [43].

### Immunohistochemical procedures

All sections for the immunohistochemistry of protein of interest were processed together. The c-fos and VIP were labeled using standard avidin-biotin protocol with minor modifications of the method described in Rastogi et al. [8], and the NPY was labeled using standard streptavidin-biotin-peroxidase protocol with minor modifications of the method described by Sakharkar et al. [44]. Each procedure began with three 10-min rinses with the PBS. Then, sections were treated for 30 min each with 0.3% hydrogen peroxide dissolved in methanol and 1% normal bovine serum albumin (BSA) dissolved in the PBS containing 0.3% Triton-X-100 (PBSBT) to block the endogenous peroxidase activity and non-specific binding, respectively. Thereafter, sections were incubated with c-fos (rabbit anti-chicken antibody; 1∶6000 in PBSBT; generously gifted by Prof. Dr. Lut Arckens, Leuven, Belgium), rabbit polyclonal VIP (1∶2500; ab43841; Abcam) or NPY (1∶6000; N9528; Sigma) antibody for 18 h at 4°C. This was followed by incubation for 2 h each with biotinylated goat anti-rabbit secondary antibody (1∶200; B2770; Invitrogen, Eugene, USA) and avidin-biotin complex (1∶110; Elite ABC Kit; Vector Laboratories, Burlingame, CA) for c-fos and VIP labeling, or with biotinylated goat anti-rabbit secondary antibody (1∶200; EXTRA-3; Sigma) and ExtrAvidin-peroxidase conjugate (1∶200; EXTRA-3; Sigma) for NPY labeling. All sections were washed with PBS and visualized for the antigen-antibody reaction by treatment for 2–3 min with diaminobenzidine solution (DAB; D4293, Sigma) prepared in 0.1M phosphate buffer (pH 7.4). After the appearance of minimal background reaction, adding more PBS stopped the color reaction. The sections were washed in the distilled water, ordered and mounted onto poly-L-lysine-coated slides, which were dehydrated in the ascending grades of alcohol, cleared in xylene and coverslipped in the DPX.

#### Antibody specificity

Control procedures were performed to verify the specificity of the immunoreaction. This included the omission of the primary antisera from the reaction as well as the replacement of the antisera with buffer or BSA. Both these procedures resulted in the total loss of immunoreactivity. Our c-fos antibody was generated in rabbit against chicken c-fos-peptide and its specificity has already been demonstrated [45]. The preadsorption tests further verified specificity of the NPY and VIP antibodies. 1 ml of diluted NPY and VIP antiserum were incubated with respective peptides (NPY - N 3266; VIP - V 3628; Sigma) at 10^−5^ M concentration for 18 h at 4°C. Incubation of brain sections with such a conjugate in place of antiserum did not yield positive immunoreaction for the NPY or VIP.

### Microscopy and data analysis

The desired image of a brain section was captured using a Leica DFC 420C digital camera attached on to Leica DM 3000 microscope. The dimension of the captured image was 2592×1944 pixel RGB 8 bit with the magnification/numerical aperture (NA): 100×/0.25 or 400×/0.65. The microscopic settings were standardized and kept constant. The images were labeled and panels were prepared using the Corel Draw X3 (Toronto, Canada).

The morphometric analysis was done using Nikon NIS-elements BR program (version 2.3). An image (100×) of the specified region was captured by a Nikon E400 microscope fixed with a DS-Fi1 Nikon digital camera in the dimension of 2560×1920 pixel RGB 8 bit, using magnification/numerical aperture (NA) at 100×/0.25. We initially standardized the microscopic setting and kept it constant. Then, immunoreactive cells were counted in the SCN for c-fos (c-fos-lir), and in the MBH for c-fos (c-fos-lir), NPY (NPY-lir) and VIP (VIP-lir). Additionally, the percent area and mean optical density of NPY-lir and VIP-lir cells in the MBH were determined.

The immunoreactive cells (ir cells) were counted as described in Rastogi et al. [8] and Kumar et al. [46]. Briefly, c-fos-lir cells were mapped in all sections containing different divisions of the SCN (mSCN and vSCN), and c-fos-, NPY- and VIP-lir cells in the MBH (DMH, IH and IN). To avoid a staining-intensity based bias in cell counting in SCN or MBH regions, we counted both types of cells, i.e. strongly (bright) and weakly (faint) stained cells [47]. Here, a subjectively defined threshold optical density, based on background staining, was used to identify the labeled cells, and cells with higher optical density than the threshold were counted as immunoreactive cells [47]. The immunoreactive cells gave mean ± SD for the group.

The cell area and mean cell optical density (OD) of the NPY- and VIP-lir cells in the MBH were quantified as described earlier for GnRH-I analysis in the preoptic area of Rufous-winged sparrows, *Aimophila carpalis* [48]. Briefly, all immunoreactive cell bodies in the different MBH regions and an out of focus image of an area of nidopallium, which is devoid of immunoreactivity, taken to indicate the background staining in areas lacking specific immunostaining, were photographed at 400×/0.65 (magnification/numerical aperture, NA). Resulting images were analyzed using Nikon NIS-elements BR program. VIP- and NPY-lir cells, excluding overlapping cell bodies, were manually outlined using a mouse-controlled cursor, and the immunoreactive area and OD (arbitrary units: 0 = no staining, complete light transmission; 1 = complete staining saturation, no light transmission) were determined for each cell, applying a 175×150 µm frame on the entire field of interest. Thus, NPY- and VIP-lir per unit field measuring 26,250 µm^2^ area were estimated from the images containing different divisions of the MBH. The immunoreactive cells above the threshold were filled with overlaid color, which gave the cell area. Subtraction of the OD of such color overlay with background image OD gave OD of the immunoreactive cell. The total area occupied by cells was calculated and percent cell area was obtained by dividing total cell area by the entire frame area. The percent cell area of images from all the sections were averaged to give a single value for each bird. Similarly, OD measurements were separately averaged. Finally, the mean ± SD for the group was calculated.

### Statistics

The data are presented as mean ± SD. We did distribution test for the data before we performed parametric test. Student t-test compared data from two groups or conditions, if it involved comparison at one time point. Two-way analysis of variance (2-way ANOVA) tested the significance of difference at two factor levels, viz. migratory/non-migratory state as factor 1, and day/night condition as factor 2. This was followed by Bonferroni post-hoc test for group comparisons, if ANOVA indicated a significant difference. A statistically significant difference was taken at *p*<0.05 level. All analyses were done using Graph Pad Prism software (version 5.0), San Diego, CA, USA.

## Results

### Experiment 1: Effect of transition from short to long day

**Figure 1:**
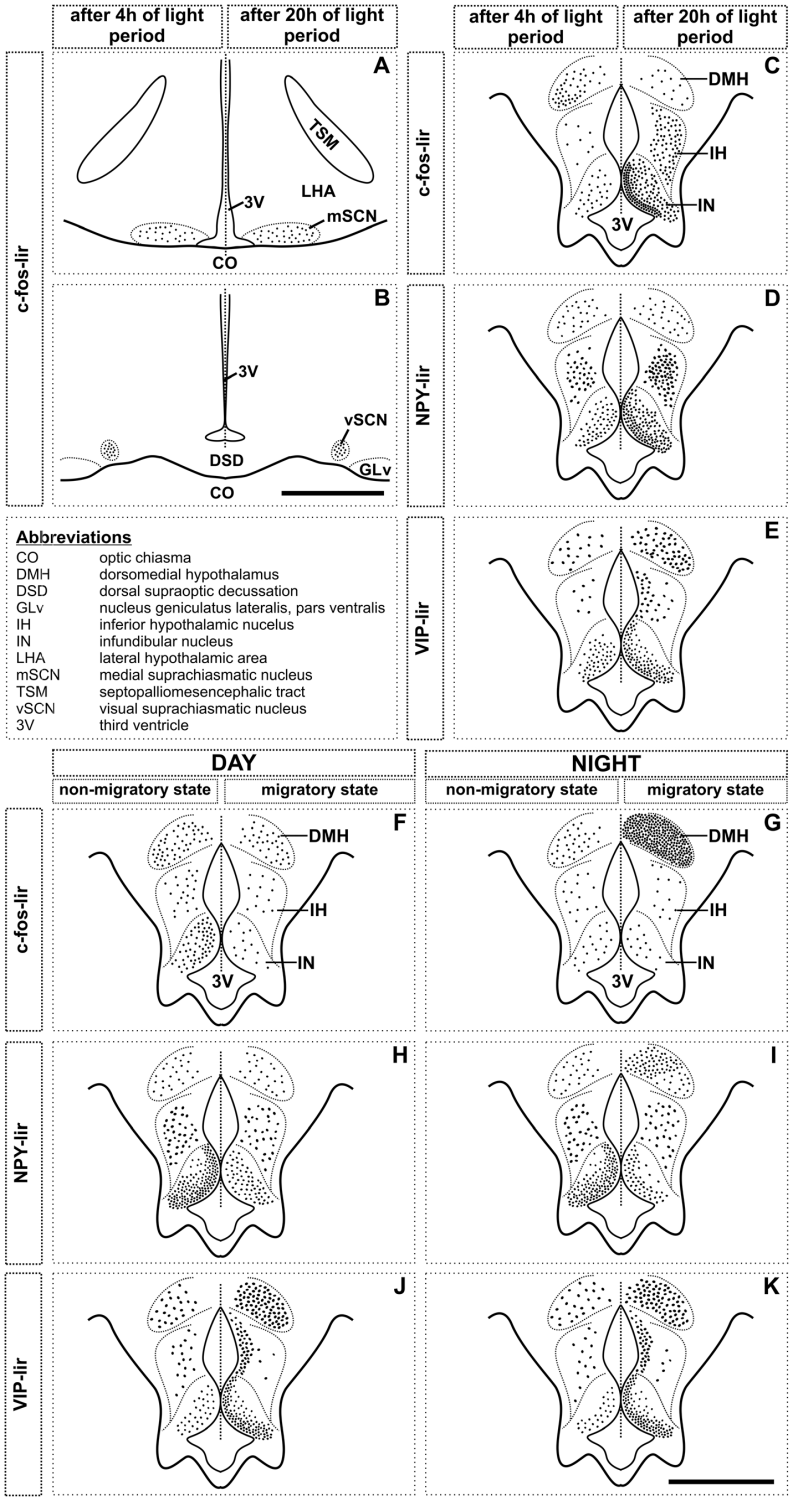
Schematic illustration of typological expression of c-fos-like immunoreactivity in suprachiasmatic nucleus (SCN), and of c-fos-, NPY- and VIP-like immunoreactivity in mediobasal hypothalamus (MBH) of blackheaded bunting. **A, B** (upper left panel)**:** c-fos immunoreactivity after 4 h (left half) or 20 h (right half) of light period on the first long day in mSCN (**A**) and vSCN (**B**). **C–E** (upper right panel)**:** c-fos (**C**), NPY (**D**) and VIP (**E**) immunoreactivity after 4 h (left half) or 20 h (right half) of light period on the first long day in the MBH. **F–K:** c-fos (**F**, **G**), NPY (**H**, **I**) and VIP (**J**, **K**) immunoreactivity in middle of the day (8 h after lights on; **F**, **H**, **J**: lower left panel) or night (4 h after lights off; **G**, **I**, **K**: lower right panel) in MBH of buntings in the non-migratory (left half) and migratory (right half) states. Scale bar = 1 mm (A, B); 500 µm (C–K). Abbreviations: 3V, third ventricle; CO, optic chiasma; DMH, dorsomedial hypothalamus; DSD, dorsal supraoptic decussation; GLv, nucleus geniculatus lateralis, pars ventralis; IH, inferior hypothalamic nucleus; IN, infundibular nucleus; LHA, lateral hypothalamic area; mSCN, medial suprachiasmatic nucleus; TSM, septopalliomesencephalic tract; vSCN, visual suprachiasmatic nucleus.

**Figure 2:**
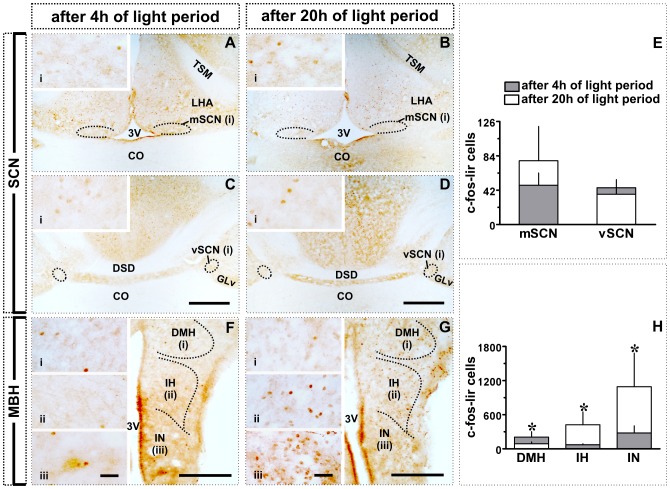
Effect of transition from short to long day in SCN and MBH: c-fos-like immunoreactivity. **A–D:** Images showing c-fos expression in mSCN and vSCN after 4 h (**A, C**) or 20 h (**B, D**) of light period. **E:** c-fos-lir cell counts (mean ± SD). **F, G:** Images showing c-fos expression in DMH, IH and IN after 4 h (**F**) or 20 h (**G**) of light period. **H:** c-fos-lir cell counts (mean ± SD). Asterisk (*) indicates significance of difference at *p*<0.05 level. Scale bar: general views = 500 µm (A–D); 200 µm (F, G); magnified views (i; ii; iii) = 25 µm. Abbreviations: 3V, third ventricle; CO, optic chiasma; DMH, dorsomedial hypothalamus; DSD; dorsal supraoptic decussation; GLv, nucleus geniculatus lateralis, pars ventralis; IH, inferior hypothalamic nucleus; IN, infundibular nucleus; LHA, lateral hypothalamic area; mSCN, medial suprachiasmatic nucleus; TSM, septopalliomesencephalic tract; vSCN, visual suprachiasmatic nucleus.

The SCN did not show significantly increased c-fos mediated neural activation in response to long days. At the end of 20 h light exposure, neither the mSCN nor vSCN had significant increase of c-fos-lir cell number as compared to ZT4 group (Fig. 1A–B, 2A–E).

Figure 1C–E schematically shows the response of MBH to 20 h light exposure (ZT20; right half) as compared to exposure to 4 h light period (ZT4; left half). In ZT20 group, c-fos-lir cells were denser in ventricular lining of the IN and dorsoventrally in the IH(Fig. 1C, right half). In this group, NPY-lir and VIP-lir cells were also denser ventricularly in the IN and medially in the IH (Figs. 1D, E). In IH, NPY-lir cells were more medial, while VIP-lir cells lined the third ventricle (Figs. 1D, E). The response of DMH was variable. c-fos-lir cells were identified in the ventrolateral portion of the DMH with less positive labeled cells in ZT20 as compared to those in ZT4 (Fig. 1 C). The numbers of NPY-lir cells were similar between these two groups (Fig. 1 D), while more VIP-lir cells were observed in ZT20 than in ZT4 group in the DMH (Fig. 1E).

**Figure 3:**
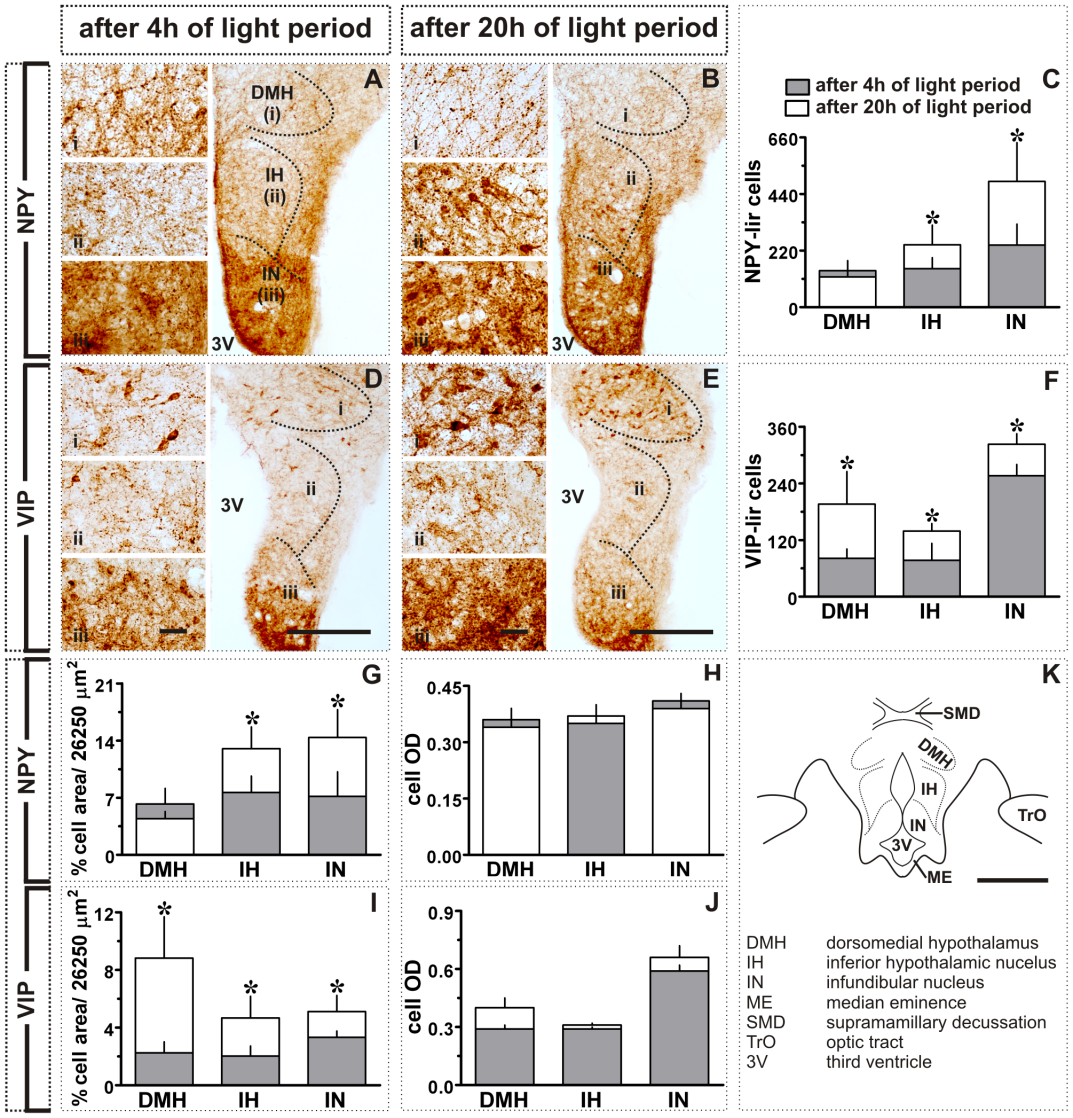
Effect of transition from short to long day in MBH: NPY- and VIP-like immunoreactivity. **A, B:** Images showing NPY expression in the DMH, IH and IN after 4(**A**) or 20 h (**B**) of light period. Also shown are the number of NPY-lir cells (mean ± SD, **C**), percent NPY-lir cell area (per grid area, 26,250 µm^2^; **G**) and mean cell optical density (cell OD; **H**). **D, E:** Images showing VIP expression in DMH, IH and IN after 4 h (**D**) or 20 h (**E**) of light period. Also shown are the number of VIP-lir cells (mean ± SD, **F**), percent VIP-lir cell area (per grid area, 26,250 µm^2^; **I**) and mean cell optical density (cell OD; **J**). **K:** Schematic illustration of different MBH subdivisions. Asterisk (*) indicates the significance of difference at *p*<0.05 level. Scale bar: general views = 200 µm (A, B, D, E); schematic (K) = 500 µm; magnified views (i - DMH; ii - IH; iii - IN) = 25 µm. Abbreviations: 3V, third ventricle; DMH, dorsomedial hypothalamus; IH, inferior hypothalamic nucleus; IN, infundibular nucleus; ME, median eminence; SMD, supramamillary decussation; TrO, optic tract.

Figure 2 shows actual (left and middle panels; Figs. 2F, G) and graphic representations (right panel; Fig. 2H) of the response of MBH to a single long day. At the end of 20 h light exposure, the number of the c-fos-lir cells was significantly increased in IH and IN than in birds sampled after 4 h of light period (*p*<0.05; Student t-test; Figs. 2F–H). In contrast, the number of c-fos-lir cells in DMH was significantly smaller in ZT20 group than in the ZT4 group (*p*<0.05; Student t-test; Figs. 2F–H). Similarly, the number of the NPY-lir cells was significantly increased in IH and IN, but not in DMH, in the ZT20 group (*p*<0.05; Figs. 3A–C). Further, as compared to ZT4 group, the number of the VIP-lir cells was significantly increased in all three MBH regions, DMH, IH and IN (*p*<0.05; Student t-test; Fig. 3D–F) at the end of 20 h light period. The data on percent cell area of NPY-lir cells paralleled the response measured in cell numbers. It was significantly increased in IH and IN (*p*<0.05; Student t-test; Fig. 3G), but not in the DMH (Fig. 3G). This was also true of the VIP immunoreactivity. As with the cell numbers, the percent cell area was significantly increased in the DMH, IH and IN (*p*<0.05; Student t-test; Fig. 3I). However, mean cell ODs for both the NPY and VIP-lir cells were not significantly different between ZT4 and ZT20 groups (Fig. 3H, J).

### Experiment 2: Effect of transition from non-migratory to migratory state

**Figure 4:**
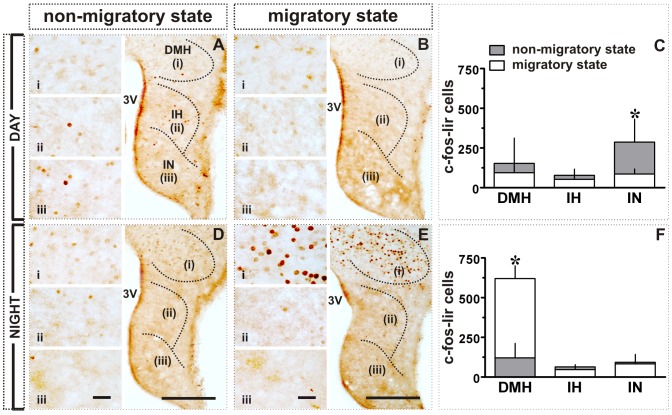
Effect of transition from non-migratory to migratory state in MBH of blackheaded bunting exposed to LD 16/8: c-fos-like immunoreactivity. **A, B, D, E:** Images showing c-fos expression in DMH, IH and IN in day (**A, B**) or night (**D, E**) during non-migratory (**A, D**) and migratory states (**B, E**). **C, F:** c-fos-lir cell numbers (mean ± SD) in DMH, IH and IN in day (**C**) or night (**F**). Asterisk (*) indicates the significance of difference at *p*<0.05 level. Scale bar: general views = 200 µm (A, B, D, E); magnified views (i - DMH; ii - IH; iii - IN) = 25 µm. Abbreviations: 3V, third ventricle; DMH, dorsomedial hypothalamus; IH, inferior hypothalamic nucleus; IN, infundibular nucleus.

**Figure 5:**
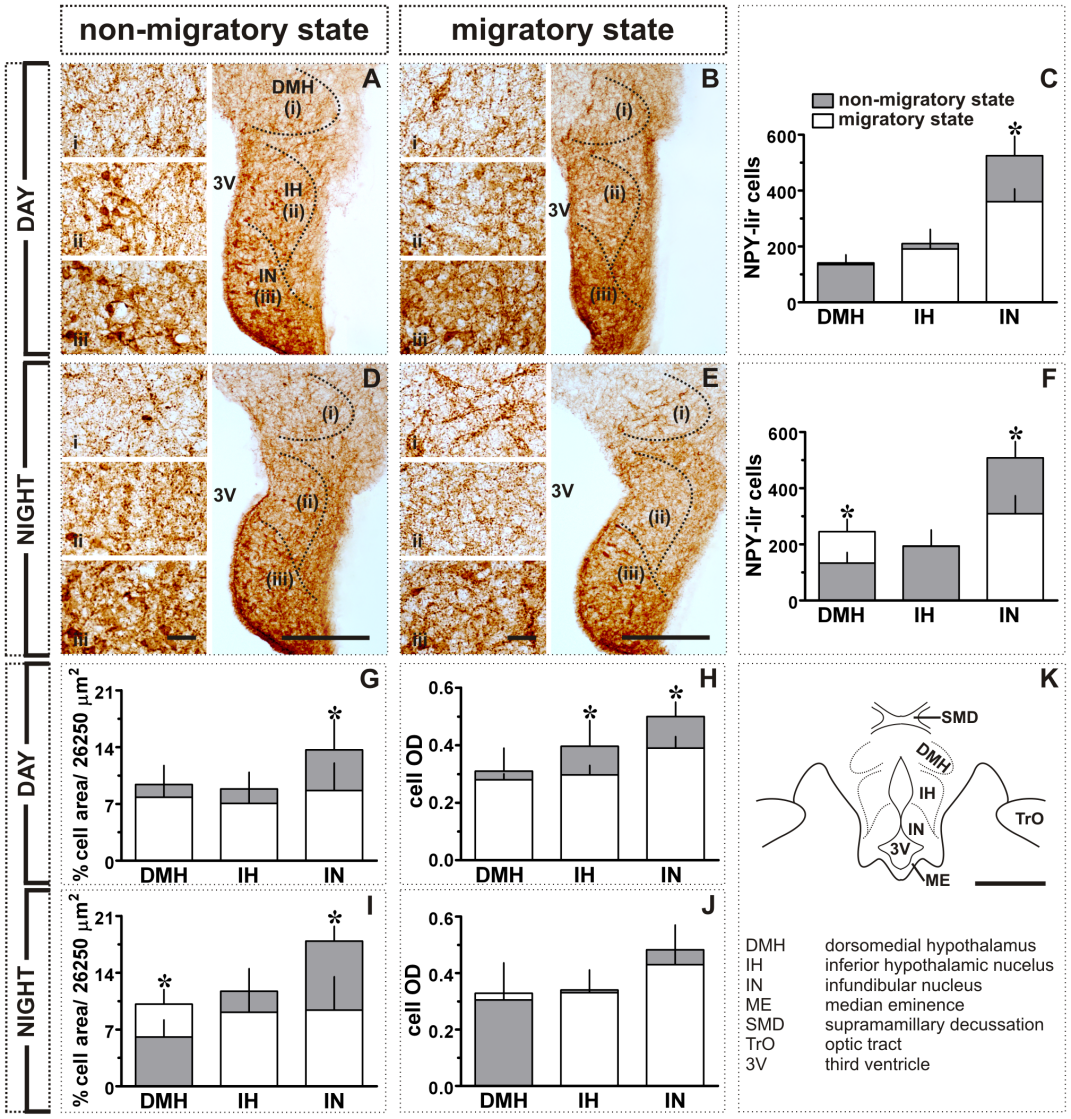
Effect of transition from non-migratory to migratory state in MBH of blackheaded bunting exposed to LD 16/8: NPY-like immunoreactivity. **A, B, D, E:** Images showing NPY expression in DMH, IH and IN in day (**A, B**) and night (**D, E**) during non-migratory (**A, D**) and migratory states (**B, E**). Also shown are NPY-lir cell numbers (mean ± SD, **C, F**), percent NPY-lir cell area (per grid area, 26, 250 µm^2^; **G, I**) and mean cell optical density (cell OD; **H, J**) in day (**C, G, H**) or night (**F, I, J**). **K:** Schematic illustration of different MBH subdivisions. Asterisk (*) indicates the significance of difference at *p*<0.05 level. Scale bar: general views = 200 µm (A, B, D, E); schematic (K) = 500 µm; magnified views (i - DMH; ii - IH; iii - IN) = 25 µm. Abbreviations: 3V, third ventricle; DMH, dorsomedial hypothalamus; IH, inferior hypothalamic nucleus; IN, infundibular nucleus; ME, median eminence; SMD, supramamillary decussation; TrO, optic tract.

**Figure 6:**
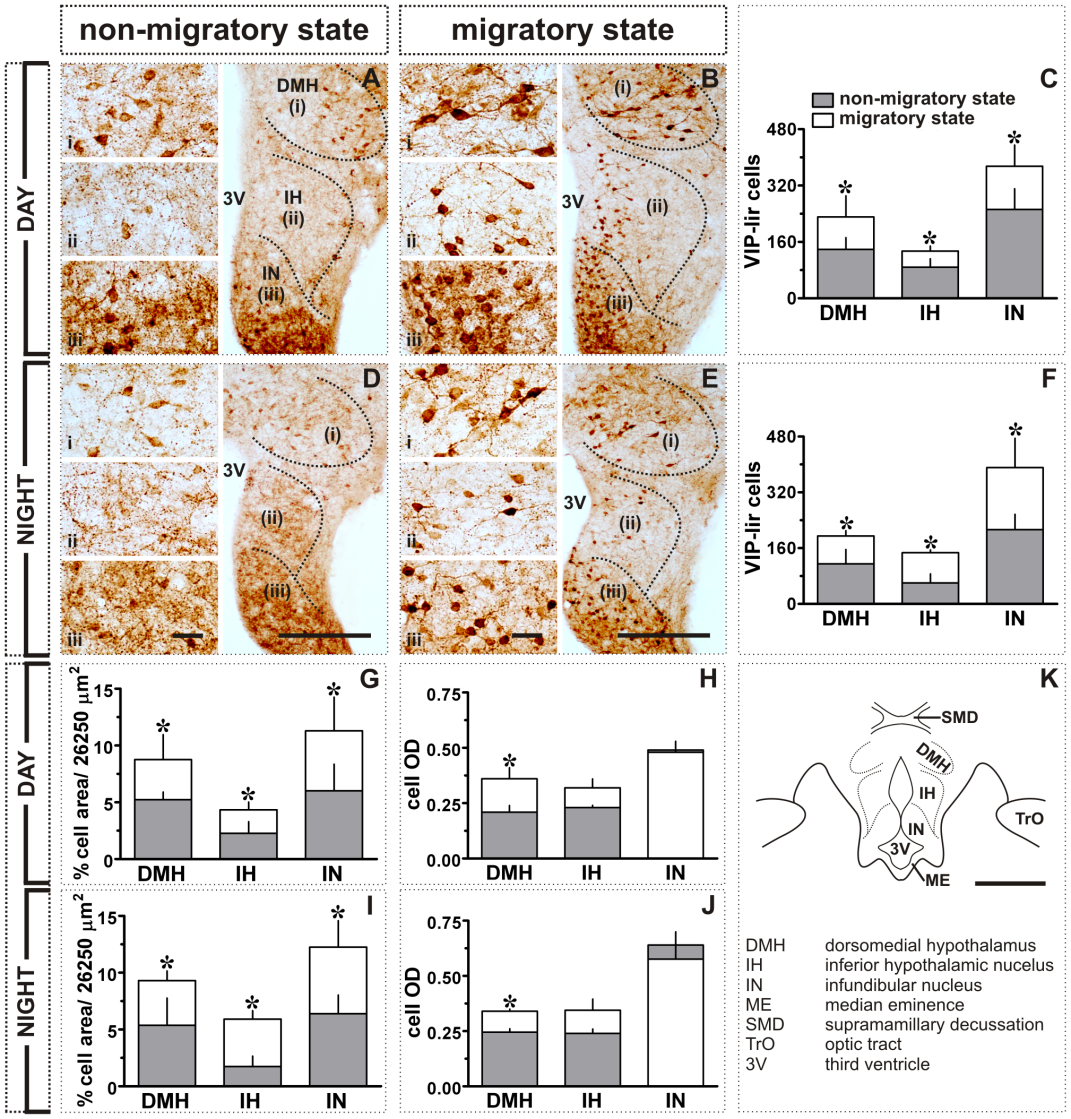
Effect of transition from non-migratory to migratory state in MBH of blackheaded bunting exposed to LD 16/8: VIP-like immunoreactivity. **A, B, D, E:** Images showing VIP expression in the DMH, IH and IN in day (**A, B**) or night (**D, E**) of the non-migratory (**A, D**) and migratory states (**B, E**). Also shown are VIP-lir cell counts (mean ± SD, **C, F**), percent NPY-lir cell area (per grid area, 26, 250 µm^2^; **G, I**) and mean cell optical density (cell OD; **H, J**) in day (**C, G, H**) or night (**F, I, J**). **K:** Schematic illustration of different MBH subdivisions. Asterisk (*) indicates the significance of difference at *p*<0.05 level. Scale bar: general views = 200 µm (A, B, D, E); schematic (K) = 500 µm; magnified views (i - DMH; ii - IH; iii - IN) = 25 µm. Abbreviations: 3V, third ventricle; DMH, dorsomedial hypothalamus; IH, inferior hypothalamic nucleus; IN, infundibular nucleus; ME, median eminence; SMD, supramamillary decussation; TrO, optic tract.

In a schematic representation, figure 1 compares the distribution of c-fos-lir (Figs. 1F, G), NPY-lir (Figs. 1H, I) and VIP-lir (Figs. 1J, K) in the subdivisions of MBH in the day (lower left panel) and night (lower right panel) between the non-migratory (left half of MBH) and migratory (right half of MBH) states. Figures 4, 5, 6 present the actual response of MBH during day and night in both the states. In general, there was a day-night difference in the expression of all three proteins in the MBH between non-migratory and migratory states, although the expressions were heterogeneous. Two way ANOVA revealed a significant difference between day and night linked with migratory states in the DMH (factor 1, migratory/non-migratory: *F*_1,19_ = 22.42, *p* = 0.0001; factor 2, day/night: *F*_1,19_ = 28.05, *p*<0.0001; interaction [factor 1×factor 2]: *F*_1,19_ = 35.99, *p* = 0.0001; Figs. 4A–F) and IN (factor 1, migratory/non-migratory: *F*_1,19_ = 7.703, *p* = 0.0121; factor 2, day/night: *F*_1,19_ = 6.686, *p* = 0.0181; interaction [factor 1×factor 2]: *F*_1,19_ = 6.306, *p* = 0.0212; Figs. 4A–F). Between two states, c-fos-lir cells were significantly densely expressed in IN during the day of the non-migratory state (*p*<0.01; Bonferroni test; Figs. 1F; 4A–C), and in DMH during the night of the migratory state (*p*<0.001; Bonferroni test; Figs. 1 G; 4D–F). Further, c-fos-lir cells were closely aligned with ventricular wall in the IN, and evenly distributed throughout in the DMH (Figs. 1F, G). There was no difference in c-fos lir cells in the IH between day and night in two behavioral states (Figs. 1F, G; 4A–F).

NPY and VIP expressions were almost antiphased. There was a significant difference in the NPY immunoreactivity between day and night linked with migratory states in the DMH (factor 1, migratory/non-migratory:*F*_1,19_ = 16.08, *p* = 0.0007; factor 2, day/night: *F*_1,19_ = 12.09, *p* = 0.0025; interaction [factor 1×factor 2]: *F*_1,19_ = 12.85, *p* = 0.0020; Figs. 5A–F). With the onset of the migratory states, the number of the NPY-lir cells was significantly increased dorsomedially in the DMH (*p*<0.001, Bonferroni test; Figs. 1 I, 5D–F) during the night, but not during the day (Figs. 1H; 5A–C). The IN had significantly higher number of the NPY-lir cells, aligned to the third ventricle (Figs. 1H, I), both in the day and night in non-migratory state than in migratory state (*p*<0.001, Bonferroni test; Figs. 5A–F). The behavioral states thus had significant effect on NPY immunoreactivity in the IN (*F*_1,19_ = 49.78, *p*<0.0001; cf. Figs. 5A–F). In IH, the number of the NPY-lir cells was not significantly different between day and night in both the states (Figs. 1H, I; 5A–F). The data on percent cell area paralleled with immunoreactive cell numbers in the MBH (Figs. 5G, I), although there were differences among the three regions. For example, in DMH, there was a significant effect of interaction, but not of the behavioral states and time of day (factor 1, migratory/non-migratory: *F*_1,19_ = 1.809, *p* = 0.1945; factor 2, day/Night: *F*_1,19_ = 0.2892, *p* = 0.5970; interaction [factor 1×factor 2]: *F*_1,19_ = 9.2670, *p* = 0.0067; cf. Figs. 5G, I). On the other hand, the IH and IN showed significant effect of only day/night and behavioral states, respectively (IH: factor 1, migratory/non-migratory - *F*_1,19_ = 3.533, *p* = 0.0756; factor 2, day/Night: F_1,19_ = 4.491, p = 0.0475; interaction [factor 1×factor 2]: *F*_1,19_ = 0.1225, *p* = 0.7301; IN: factor 1, migratory/non-migratory: *F*_1,19_ = 20.29, *p* = 0.0002; factor 2, day/Night: *F*_1,19_ = 2.760, *p* = 0.1130; interaction [factor 1×factor 2]: *F*_1,19_ = 1.363, *p* = 0.2574; cf. Figs. 5G, I). Further, mean cell optical density (OD) of NPY-lir cells in the day was significantly reduced in IH and IN in the migratory state as compared to the non-migratory state (*p*<0.05, Bonferroni test; Fig. 5H), but cell OD was not changed in the DMH (Fig. 5H). The cell OD at night was not affected in all the three MBH regions (Fig. 5J).

VIP immunoreactivity, as measured in VIP-lir cell numbers, was significantly increased both in the day and night in all three MBH regions, DMH, IH and IN, in migratory state as compared to the non-migratory state (*p*<0.05, Bonferroni test; Figs. 1J, K; 6A–F). There was a significant effect of behavioral states on VIP immunoreactivity in the MBH regions (DMH: *F*_1,10_ = 16.19, *p* = 0.0024; IH: *F*_1,10_ = 35.95, *p* = 0.0001; IN: *F*_1,10_ = 20.79, *p* = 0.0010; two-way ANOVA; cf. Figs. 6A–F).VIP-lir cells in IH and IN lined the third ventricle, but they were distributed throughout in the DMH (Fig. 1J, K). The data on the percent cell area paralleled with labeled cell numbers (*p*<0.05, Bonferroni test; Figs. 6G, I) and showed significant effect of the migratory state (DMH: *F*_1,10_ = 16.03, *p* = 0.0025; IH: *F*_1,10_ = 43.06, *p*<0.0001; IN: *F*_1,10_ = 20.11, *p* = 0.0012; two-way ANOVA; cf. Figs. 6G, I). Further, mean cell OD did not change in IH or IN, except in the day and night in DMH when OD was significantly increased (*p*<0.05, Bonferroni test; Figs. 6H, J) in the migratory state. Two-way ANOVA further showed a significant effect of behavioral states in cell OD in the DMH (*F*_1,10_ = 22.68, *p* = 0.0008; cf. Figs. 6H, J) and IH (*F*_1,10_ = 7.913, *p* = 0.0184; cf. Figs. 6H, J).

## Discussion

The lack of a significant increase in the number of the c-fos-lir cells in SCN after 20 h light period may suggest the absence of the direct role of SCN in the photoperiodic induction of seasonal responses in the blackheaded bunting (Figs. 1A–B; 2A–E), as reported in turkeys [12]. We would like to interpret this as evidence for the independence of the SCN activity from photoperiodic conditions to which birds are exposed to in the year. This interpretation is consistent with the absence of a daily rhythm in c-fos-lir cells in the SCN of chicken, Japanese quail, European starling and house sparrow kept on long days [49]–[51].

A 4–5 fold increase in the number of the c-fos-lir cells occurred in the ventral division of MBH (IH and IN) of buntings exposed to a 20 h light period (Figs. 1C; 2F–H), as is shown in levels of *c-fos* mRNA and protein in MBH of other birds on the first day of exposure to long days [12], [16]. Therefore, IH and IN appear to be the probable photoperiodic induction sites in the long day breeding birds [16]–[20]. We cannot rule out nonetheless, the possibility of the c-fos expression in MBH as the consequence of activation of another parallel neural mechanism on exposure of birds to long days.

Conversely, c-fos expression was not enhanced on the first long day in DMH; in fact, the number of the c-fos-lir cells was significantly high at ZT4 than at ZT20 (*p*<0.05; Figs. 1C, 2F–H). However, DMH had increased number of the c-fos-lir cells at night in the migratory state (Experiment 2; cf. Figs. 1G; 4D–F). Was this the consequence of the induction of *Zugunruhe*, an inversed active state in night migrants during migration? It is known in mammals that DMH afferents project to areas that are critical in maintaining brain in an active state [52], [53]. In rats, for example, SCN-DMH circuit that regulates sleep-wake cycle [54] has a higher c-fos induction during wakefulness [55]. We would therefore, argue that DMH is possibly involved in regulation of heightened daily activities like locomotion and vigilance states during the migratory state in buntings. Could then DMH be considered as the site of functional integration between central and peripheral clocks involved in the regulation of migration? This needs to be investigated.

In close association with c-fos-lir cells, the numbers of the NPY- and VIP-lir cells were significantly enhanced in MBH of buntings on the first long day (cf. Figs. 1D, E; 3A–F). Elevated VIP [21], [26] and NPY [33] levels have been reported also in other birds kept under long days. This may suggest a close functional association between photoperiodic neural systems. A close anatomical association of VIP and NPY with GnRH neurons has been described under long days in few birds [26], [30], [56]. We would argue that VIP and NPY are involved in the photoperiodic regulation of seasonal events in birds including blackheaded buntings, in several possible ways. VIP neurons act as encephalic photoreceptors - EPRs [21], [24], [57], which on photostimulation release VIP and modulate the activity of the photoneuroendocrine axis [21]. VIP may also induce the expression of type 2 deiodinase (*Dio2*, the enzyme that converts thyroxin (T4) into active iodothyronine (T3), [58]) and, in turn, results in testicular growth under long days [20]. *Dio2* levels are significantly increased in the MBH of Japanese quail exposed to a single long day [20]. Further, VIP stimulates the synthesis and release of prolactin [28], [59], [60] that may influence photoperiod induced gonadal maturation in buntings [61].

Between non-migratory and migratory states, VIP and NPY expressions were antiphased, regardless of the day or night condition (cf. Figs. 1H–I, J–K; 5A–F; 6A–F). Buntings had high NPY in the non-migratory state (Figs. 1H–I), while high VIP in the migratory state (Figs. 1J–K). A high NPY in the non-migratory state possibly reflects change in the sensitivity of feeding response (increased food intake) and the initiation of hypothalamo-pituitary-gonad (HPG) axis activation in buntings at the end of the first seven long days [31], [33], [34], [62]. Similarly, higher VIP in the migratory state suggests an enhanced light sensitivity of the EPRs, probably required during the night migration. Notwithstanding, however, both VIP and NPY were increased at night in the migratory state in the DMH (cf. Figs. 1I, K; 5D–F; 6D–F). We discount the possibility of increased NPY levels in DMH as the consequence of an increased night activity, as has been suggested for high NPY in DMH during increased physical activity at night in rats [63]. This is because NPY-lir cells were still low in number during the day of the non-migratory state when buntings were predominantly day active (Figs. 1H, 5A–C). At present, however, it remains arguable whether observed differences in neural changes between non-migratory and migratory states were due to the induction of migration alone, or due to also different extent of the HPG axis activation under long days after seven days and after seven days of *Zugunruhe*. By about three weeks of exposure to long days, when buntings have shown seven days of *Zugunruhe*, birds will have recrudesced testes [8]. But, blackheaded buntings in the experiment 2 indeed presented distinct migratory phenotypes between their non-migratory and migratory states, with the latter characterized by the phase-inversed activity behavior [8]. Also testes are not involved in the photoperiodic induction of migration in the blackheaded bunting [64], [65].

In conclusion, SCN does not appear to be the site of photoperiod-induced seasonal responses in buntings. We suggest that ventral division of MBH (IH and IN) in buntings contains the seasonal clock, which is sensitive to annual photoperiodic changes. VIP and NPY are the parts of the neuroendocrine mechanism(s) involved, respectively, in sensing light and translating photoperiodic message into a neuroendocrine response. Further, we would like to propose the role of the dorsal division of the MBH (DMH) in the night-time migration. An increased neural activity in DMH at night parallels nocturnal shift of the activity behavior in buntings with the onset of the migration [8]. This may be taken to suggest DMH as the possible site of the functional coupling between the endogenous clock and behavioral output, e.g. *Zugunruhe*. Overall, these are the first results in showing the involvement of MBH with region-specific roles in the regulation of photoperiod induced seasonal events in a night-migratory songbird.

## References

[1] Gwinner E (1996) Circadian and circannual programmes in avian migration. J Exp Biol 199: 39–48. 9317295 10.1242/jeb.199.1.39

[2] Kumar V, Singh BP, Rani S (2004) The Bird Clock: A complex multi oscillatory and highly diversified system. Biol Rhythm Res 35: 121–144.

[3] Dawson A, King VM, Bentley GE, Ball GF (2001) Photoperiodic control of seasonality in birds. J Biol Rhythms 16: 365–380. 11506381 10.1177/074873001129002079

[4] Kumar V, Wingfield JC, Dawson A, Ramenofsky M, Rani S, et al (2010) Biological clocks and regulation of seasonal reproduction and migration in birds. Physiol Biochem Zool 83: 827–835. 20604684 10.1086/652243

[5] Budki P, Rani S, Kumar V (2012) Persistence of circannual rhythms under constant periodic and aperiodic light conditions: sex differences and relationship with the external environment. J Exp Biol 215: 3774–3785. 22811243 10.1242/jeb.065581

[6] Bartell P, Gwinner E (2005) A separate circadian oscillator controls nocturnal migratory restlessness in a songbird *Sylvia borin* . J Biol Rhythms 20: 538–549. 16275772 10.1177/0748730405281826

[7] Rani S, Malik S, Trivedi AK, Singh S, Kumar V (2006) A circadian clock regulates migratory restlessness in the blackheaded bunting, *Emberiza melanocephala* . Curr Sci 91: 1093–1096.

[8] Rastogi A, Kumari Y, Rani R, Kumar V (2011) Phase inversion of neural activity in the olfactory and visual systems of a night-migratory bird during migration. Eur J Neurosci 34: 99–109. 21676040 10.1111/j.1460-9568.2011.07737.x

[9] Sumova A, Jac M, Sladek M, Illnerova H (2002) Entrainment of the circadian pacemaker in the rat suprachiasmatic nucleus. Physiol Res 51: 44P. 10.1016/s0006-8993(02)02933-5 12176169

[10] Cantwell EL, Cassone VM (2006) Chicken suprachiasmatic nuclei: I. efferent and afferent connections. J Comp Neurol 496: 97–120. 16528725 10.1002/cne.20935 PMC2590781

[11] Wilson FE (1991) Neither retinal nor pineal photoreceptors mediate photoperiodic control of seasonal reproduction in American tree sparrows (*Spizella arborea*). J Exp Zool 259: 117–127.

[12] Thayananuphat A, Kang SW, Bakken T, Millam JR, El Halawani ME (2007) Rhythm-dependent light induction of the c-fos gene in the turkey hypothalamus. J Neuroendocrinol 19: 407–417. 17388817 10.1111/j.1365-2826.2007.01544.x

[13] Kumar V (1988) Investigations of photoperiodically induced fattening in migratory blackheaded bunting (*Emberiza melanocephala*) (Aves). J Zool Lond 216: 253–263.

[14] Sharp PJ, Follett BK (1969) The effect of hypothalamic lesions on gonadotrophin release in Japanese quail (*Coturnix coturnix japonica*). Neuroendocrinology 5: 205–218. 5362399 10.1159/000121861

[15] Ohta M, Wada M, Homma K (1984) Induction of rapid testicular growth in Japanese quail by phasic electrical stimulation of the hypothalamic photosensitive area. J Comp Physiol A 154: 583–589.

[16] Meddle SL, Follett BK (1997) Photoperiodically driven changes in Fos expression within the basal tuberal hypothalamus and median eminence of Japanese quail. J Neurosci 17: 8909–8918. 9348357 10.1523/JNEUROSCI.17-22-08909.1997 PMC6573072

[17] Yasuo S, Watanabe M, Okabayashi N, Ebihara S, Yoshimura T (2003) Circadian clock genes and photoperiodism: comprehensive analysis of clock gene expression in the mediobasal hypothalamus, the suprachiasmatic nucleus, and the pineal gland of Japanese quail under various light schedules. Endocrinology 144: 3742–3748. 12933643 10.1210/en.2003-0435

[18] Nakao N, Ono H, Yamamura T, Anraku T, Takagi T, et al (2008) Thyrotrophin in the pars tuberalis triggers photoperiodic response. Nature 452: 317–U311. 18354476 10.1038/nature06738

[19] Stevenson TJ, Ball GF (2012) Disruption of neuropsin mRNA expression via RNA interference facilitates the photoinduced increase in thyrotropin-stimulating subunit b in birds. Eur J Neurosci 36: 2859–2865 10.1111/j.1460-9568.2012.08209.x 22775245

[20] Yoshimura T, Yasuo S, Watanabe M, Iigo M, Yamamura T, et al (2003) Light-induced hormone conversion of T4 to T3 regulates photoperiodic response of gonads in birds. Nature 426: 178–181. 14614506 10.1038/nature02117

[21] Li H, Kuenzel WJ (2008) A possible neural cascade involving the photoneuroendocrine system (PNES) responsible for regulating gonadal development in an avian species, *Gallus gallus* . Brain Res Bul 76: 586–596. 10.1016/j.brainresbull.2008.04.007 18598849

[22] Perfito N, Jeong SY, Silverin B, Calisi RM, Bentley GE, et al (2012) Anticipating Spring: Wild Populations of Great Tits (*Parus major*) Differ in Expression of Key Genes for Photoperiodic Time Measurement. PLoS ONE 7: e34997 10.1371/journal.pone.0034997 22539953 PMC3334499

[23] Saab SS, Lange HS, Maney DL (2010) Gonadotrophin-releasing hormone neurons in a photoperiodic songbird express Fos and Egr-1 protein after a single long day. J Neuroendocrinol 22: 196–207. 20070482 10.1111/j.1365-2826.2010.01954.x

[24] Silver R, Witkovsky P, Horvath P, Alones V, Barnstable CJ, et al (1988) Coexpression of opsin- and VIP-like-immunoreactivity in CSF-contacting neurons of the avian brain. Cell Tis Res 253: 189–198. 10.1007/BF00221754 2970894

[25] Rathinam T, Kuenzel WJ (2005) Attenuation of gonadal response to photostimulation following ablation of neurons in the lateral septal organ of chicks. Brain Res Bull 64: 455–461. 15607834 10.1016/j.brainresbull.2004.10.003

[26] Teruyama R, Beck MM (2001) Double immunocytochemistry of vasoactive intestinal peptide and cGnRH-I in male quail: photoperiodic effects. Cell Tis Res 303: 403–414. 10.1007/s004410000313 11320656

[27] Proudman JA, Opel H (1983) Stimulation of prolactin and growth hormone secretion from turkey pituitary cells. Poult Sci 62: 1484–1485.

[28] Macnamee MC, Sharp PJ, Lea RW, Sterling RJ, Harvey S (1986) Evidence that vasoactive intestinal polypeptide is a physiological prolactin-releasing factor in the bantam hen. Gen Comp Endocrinol 62: 470–478. 3770438 10.1016/0016-6480(86)90057-2

[29] Sharp PJ, Sterling RJ, Talbot RT, Huskisson NS (1989) The role of hypothalamic vasoactive intestinal polypeptide in the maintenance of prolactin secretion in incubating bantam hens: observations using passive immunization, radioimmunoassay and immunohistochemistry. J Endocrinol 122: 5–13. 2769162 10.1677/joe.0.1220005

[30] Kuenzel WJ (2000) Central nervous system regulation of gonadal development in the avian male. Poult Sci 79: 1679–1688. 11092343 10.1093/ps/79.11.1679

[31] Contijoch AM, Malamed S, McDonald JK, Advis JP (1993) Neuropeptide Y regulation of LHRH release in the median eminence: immunocytochemical and physiological evidence in hens. Neuroendocrinology 57: 135–145. 8479609 10.1159/000126353

[32] Fraley GS, Kuenzel WJ (1993) Precocious puberty in chicks (*Gallus domesticus*) induced by central injections of neuropeptide Y. Life Sci 52: 1649–1656. 8483393 10.1016/0024-3205(93)90047-7

[33] Richardson RD, Boswell T, Raffety BD, Seeley RJ, Wingfield JC, et al (1995) NPY increases food intake in white-crowned sparrows: effect in short and long photoperiods. Am J Physiol 268: R1418–R1422. 7611518 10.1152/ajpregu.1995.268.6.R1418

[34] Boswell T (2005) Regulation of energy balance in birds by the neuroendocrine hypothalamus. J Poult Sci 42: 161–181.

[35] Ramakrishnan S, Strader AD, Wimpee B, Chen P, Smith MS, et al (2007) Evidence for increased neuropeptide Y synthesis in mediobasal hypothalamus in relation to parental hyperphagia and gonadal activation in breeding ringdoves. J Neuroendocrinol 19: 163–171. 17280589 10.1111/j.1365-2826.2006.01520.x

[36] Mouritsen H, Feenders G, Liedvogel M, Wada K, Jarvis ED (2005) Night-vision brain area in migratory songbirds. Proc Natl Acad Sci USA 102: 8339–8344 doi 10.1073pnas.0409575102/pnas.0409575102 15928090 10.1073/pnas.0409575102 PMC1149410

[37] Zapka M, Heyers D, Hein CM, Engels S, Schneider NL, et al (2009) Visual but not trigeminal mediation of magnetic compass information in a migratory bird. Nature 461: 1274–1277. 19865170 10.1038/nature08528

[38] Heyers D, Zapka M, Hoffmeister M, Wild JM, Mouritsen H (2010) Magnetic field changes activate the trigeminal brainstem complex in a migratory bird. Proc Natl Acad Sci USA 107: 9394–9399 doi/10.1073/pnas.0907068107. 20439705 10.1073/pnas.0907068107 PMC2889125

[39] Kumar V, Jain N, Singh BP, Kumar BS (1993) Plasma levels of luteinizing hormone in intact and castrated blackheaded bunting *(Emberiza melanocephala)* exposed to stimulatory and nonstimulatory photoperiods. Reprod Nutr Dev 33: 143–150. 8363738 10.1051/rnd:19930207

[40] Ali S, Ripley SD (1974) Handbook of birds of India and Pakistan. Bombay: Oxford University Press.

[41] Malik S, Rani S, Kumar V (2004) Wavelength dependency of light-induced effects on photoperiodic clock in the migratory blackheaded bunting (*Emberiza melanocephala*). Chronobiol Int 21: 367–384. 15332443 10.1081/cbi-120038742

[42] Klüver H, Barrera E (1953) A method for the combined staining of cells and fibers in the nervous system. J Neuropathol Exp Neurol 12: 400–403. 13097193 10.1097/00005072-195312040-00008

[43] Sharp PJ (2005) Photoperiodic regulation of seasonal breeding in birds. Annals of the New York Academy of Sciences 1040: 189–199. 15891024 10.1196/annals.1327.024

[44] Sakharkar AJ, Singru PS, Sarkar K, Subhedar N (2005) Neuropeptide Y in the forebrain of the adult male cichlid fish *Oreochromis mossambicus*: distribution, effects of castration and testosterone replacement. J Comp Neurol 489: 148–165. 15984003 10.1002/cne.20614

[45] D'hondt E, Vermeiren J, Peeters K, Balthazart J, Tlemcani O, et al (1999) Validation of a new antiserum directed towards the synthetic c-terminus of the FOS protein in avian species: immunological, physiological and behavioral evidence. J Neurosci Methods 91: 31–45. 10522822 10.1016/s0165-0270(99)00067-9

[46] Kumar V, Goguen DM, Guido ME, Rusak B (1997) Melatonin does not influence the expression of c-fos in the suprachiasmatic nucleus of rats and hamsters. Mol Brain Res 52: 242–248. 9495545 10.1016/s0169-328x(97)00260-x

[47] Gentner TQ, Hulse SH, Duffy D, Ball GF (2001) Response biases in auditory forebrain regions of female songbirds following exposure to sexually relevant variation in male song. J Neurobiol 46: 48–58. 11108615 10.1002/1097-4695(200101)46:1<48::aid-neu5>3.0.co;2-3

[48] Small TW, Sharp PJ, Bentley GE, Millar RP, Tsutsui K, et al (2008) Photoperiod-independent hypothalamic regulation of luteinizing hormone secretion in a free-living Sonaran desert bird, the Rufous-winged sparrow (*Aimophila carpalis*). Brain Behav Evol 71: 127–142. 18032888 10.1159/000111459

[49] King VM, Follett BK (1997) c-fos expression in the putative avian suprachiasmatic nucleus. J Comp Physiol A 180: 541–551. 9163930 10.1007/s003590050071

[50] Wallman J, Saldanha CJ, Silver R (1994) A putative suprachiasmatic nucleus of birds responds to visual motion. J Comp Physiol A 174: 297–304. 8151521 10.1007/BF00240212

[51] Brandstätter R, Abraham U (2003) Hypothalamic circadian organization in birds. I. Anatomy, functional morphology, and terminology of the suprachiasmatic region. Chronobiol Int 20: 637–655. 12916717 10.1081/cbi-120023343

[52] Bernardis LL, Bellinger LL (1998) The dorsomedial hypothalamic nucleus revisited: update. Proc Soc Exp Biol Med 218: 284–306. 9714072 10.3181/00379727-218-44296

[53] Chou TC, Scammell TE, Gooley JJ, Gaus SE, Saper CB, et al (2003) Critical role of dorsomedial hypothalamic nucleus in a wide range of behavioral circadian rhythms. J Neurosci 23: 10691–10702. 14627654 10.1523/JNEUROSCI.23-33-10691.2003 PMC6740926

[54] Aston-Jones G, Chen S, Zhu Y, Oshinsky ML (2001) A neural circuit for circadian regulation of arousal. Nat Neurosci 4: 732–738. 11426230 10.1038/89522

[55] Saper CB, Lu J, Chou TC, Gooley J (2005) The hypothalamic integrator for circadian rhythms. Trends Neurosci 28: 152–157. 15749169 10.1016/j.tins.2004.12.009

[56] Saldanha CJ, Silverman AJ, Silver R (2001) Direct innervation of GnRH neurons by encephalic photoreceptors in birds. J Biol Rhythms 16: 39–49. 11220777 10.1177/074873040101600105 PMC3281767

[57] Wang G, Wingfield JC (2011) Immunocytochemical study of rhodopsin-containing putative encephalic photoreceptors in house sparrow, *Passer domesticus* . Gen Comp Endocrinol 170: 589–596. 21118688 10.1016/j.ygcen.2010.11.014

[58] Molinero P, Guerrero JM (1993) Vasoactive intestinal peptide stimulates type II thyroxine 5-deiodinase and *N*-acetyltransferase activities in dispersed pineal cells of euthyroid and hypothyroid rats. Neurosci Lett 151: 130–133. 8506071 10.1016/0304-3940(93)90003-4

[59] El Halawani ME, Youngren OM, Pitts GR (1997) Vasoactive intestinal peptide as the avian prolactin releasing factor. In: Harvey S, Etches RJ, editors. Perspectives in Avian Endocrinology. Bristol: Journal of Endocrinology Ltd. pp. 403–416.

[60] Opel H, Proudman JA (1988) Stimulation of prolactin release in turkeys by vasoactive intestinal peptide. Proc Soc Exp Biol Med 187: 455–460. 3353394 10.3181/00379727-187-42688

[61] Tewary PD, Kumar V, Tripathi BK (1984) Response to exogenous prolactin during gonadal photostimulation in blackheaded bunting. Current Sci 53: 1307–1308.

[62] Kuenzel WJ, Douglass LW, Davison BA (1987) Robust feeding following central administration of neuropeptide Y or peptide YY in chicks, *Gallus domesticus* . Peptides 8: 823–828. 3432131 10.1016/0196-9781(87)90066-0

[63] Kawaguchi M, Scott KA, Moran TH, Bi S (2005) Dorsomedial hypothalamic corticotropin-releasing factor mediation of exercise-induced anorexia. Am J Physiol Regul Integr Comp Physiol 288: R1800–R1805. 15677523 10.1152/ajpregu.00805.2004

[64] Tewary PD, Kumar V (1981) Effect of castration on photoperiodically induced weight gain in a migratory finch: Blackheaded bunting *Emberiza melanocephala* . Indian J Exp Biol 19: 469–471.

[65] Gupta NJ, Kumar V (2013) Testes play a role in termination but not in initiation of the spring migration in the night-migratory blackheaded bunting. Anim Biol (in press).

